# Central Noradrenergic Neurotransmission and Weight Loss 6 Months After Gastric Bypass Surgery in Patients with Severe Obesity

**DOI:** 10.1007/s11695-021-05657-7

**Published:** 2021-08-19

**Authors:** J. Marvin Soeder, Julia Luthardt, Michael Rullmann, Georg A. Becker, Mohammed K. Hankir, Marianne Patt, Philipp M. Meyer, Tatjana Schütz, Yu-Shin Ding, Anja Hilbert, Arne Dietrich, Osama Sabri, Swen Hesse

**Affiliations:** 1 Integrated Research and Treatment Center (IFB) Adiposity Diseases, Leipzig University Medical Centre, 04103 Leipzig, Germany; 2 Department of Nuclear Medicine, University Hospital Leipzig, 04103 Leipzig, Germany; 3 Department of Experimental Surgery, University Hospital Würzburg, 97080 Würzburg, Germany; 4 Departments of Radiology and Psychiatry, New York University School of Medicine, New York City, NY 10016 USA; 5 Department of Medical Psychology and Medical Sociology and Department of Psychosomatic Medicine and Psychotherapy, University Hospital Leipzig, 04103 Leipzig, Germany; 6 Department of Visceral, Transplant, Thoracic and Vascular Surgery, Bariatric Surgery Section, University Hospital Leipzig, 04103 Leipzig, Germany

**Keywords:** RYGB, Obesity, MRB, Noradrenaline, Noradrenaline transporter, PET

## Abstract

**Purpose:**

Roux-en-Y gastric bypass (RYGB) surgery is currently the most efficient treatment to achieve long-term weight loss in individuals with severe obesity. This is largely attributed to marked reductions in food intake mediated in part by changes in gut-brain communication. Here, we investigated for the first time whether weight loss after RYGB is associated with alterations in central noradrenaline (NA) neurotransmission.

**Materials and Methods:**

We longitudinally studied 10 individuals with severe obesity (8 females; age 43.9 ± 13.1 years; body mass index (BMI) 46.5 ± 4.8 kg/m^2^) using (S,S)-[^11^C]O-methylreboxetine and positron emission tomography to estimate NA transporter (NAT) availability before and 6 months after surgery. NAT distribution volume ratios (DVR) were calculated by volume-of-interest analysis and the two-parameter multilinear reference tissue model (reference region: occipital cortex).

**Results:**

The participants responded to RYGB surgery with a reduction in BMI of 12.0 ± 3.5 kg/m^2^ (*p* < 0.001) from baseline. This was paralleled by a significant reduction in DVR in the dorsolateral prefrontal cortex (pre-surgery 1.12 ± 0.04 vs. post-surgery 1.07 ± 0.04; *p* = 0.019) and a general tendency towards reduced DVR throughout the brain. Furthermore, we found a strong positive correlation between pre-surgery DVR in hypothalamus and the change in BMI (*r* = 0.78; *p* = 0.01).

**Conclusion:**

Reductions in BMI after RYGB surgery are associated with NAT availability in brain regions responsible for decision-making and homeostasis. However, these results need further validation in larger cohorts, to assess whether brain NAT availability could prognosticate the outcome of RYGB on BMI.

**Graphical abstract:**

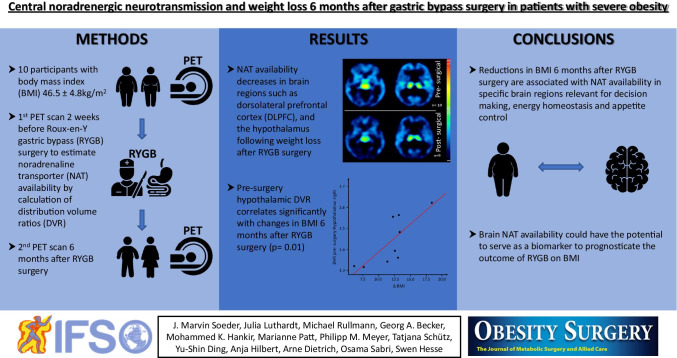

## Introduction

According to recent estimates, as much as 39% of adults globally are overweight and 13% are obese, with these figures expected to rise even further in the future [1]. The negative consequences of overweight and obesity to health are serious and include the development of chronic debilitating diseases such as type 2 diabetes, hypertension, and various cancers [2], which place a tremendous strain on health care systems [3]. Therefore, research into and the development of sustainable treatment regimens for obesity and especially severe obesity with body mass index (BMI) > 35 kg/m^2^ are urgently needed.

Currently, bariatric surgery, especially Roux-en-Y gastric bypass (RYGB), is the most efficient treatment to achieve significant long-term weight loss in individuals with severe obesity along with amelioration of their comorbidities [4]. The persistently negative energy balance following RYGB is considered to result from a combination of reduced energy intake and increased energy expenditure through complex changes in physiology and not merely mechanical effects such as malabsorption and the restricted stomach size [5–7]. Indeed, evidence from various functional magnetic resonance imaging (fMRI) studies suggests that RYGB uniquely modifies gut-brain communication to favorably impact on the activity of homeostatic, reward, and executive brain feeding areas, resulting in altered food preference [8]. Most recently, RYGB patients who showed the greatest changes in taste-induced activation of the ventral tegmental area (VTA), a major brain reward area, 2 weeks post-surgery exhibited the greatest weight loss [9]. Further, previous positron emission tomography (PET) imaging studies have been performed on how RYGB affects important feeding-regulatory neurotransmitter systems in homeostatic, reward, and executive brain areas showing that brain opioid receptor expression is normalized post-surgery [10] and that changes in neocortical 5-HT_2A_ receptors associate with changes in BMI [11]. Collectively, these findings suggest that neuroimaging (particularly molecular neuroimaging) studies of patients with RYGB may not only help guide the formulation of novel centrally acting weight loss drugs, but may also provide predictive information on which patients respond most favorably to RYGB in terms of weight loss.

The central noradrenaline (NA) system is strongly involved in regulating mood and feeding behavior, with its main source in the locus coeruleus (LC) of the dorsal tegmentum of the pons [12]. The LC sends extensive efferent NA projections throughout the cerebral cortex but also to the hypothalamus. We have previously shown that the lowered hypothalamic NAT availability correlates positively with strengthened connectivity between hypothalamus and insula, known as the primary gustatory cortex and medial orbitofrontal cortex (OFC), the key area for global judgement of food evaluation and decision-making, using the highly NA transporter (NAT)-selective PET radiotracer (S,S)-[^11^C]O-methylreboxetine ([^11^C]MRB) [13–15]. These findings point towards the NA control of hypothalamic function in maintaining energy homeostasis and the regulation of hunger and satiety. A direct relationship, however, between the NA system and changes in body weight (BW) following surgical intervention, i.e., RYGB, has not yet been explored. Based on own previous studies with dietary intervention and a normal weight control group [16, 17] and a study by Li et al. [18], our general assumption is that NAT availability as a marker of the central noradrenergic tone significantly decreases with reduction in BMI from severe (BMI > 35 kg/m^2^) to moderate obesity (BMI 30–35 kg/m^2^) but increases with reduction from moderate obesity to normal body weight (BMI 18.5–25 kg/m^2^) describing a U-shaped curve, specifically in the hypothalamus, the insula, and the prefrontal brain area.

The aim of this proof-of-concept study was to investigate (I) whether RYGB surgery and consequent BW loss have an effect on the central noradrenergic system and (II) whether the changes in BMI and changes in BW are predictable by pre-surgical NAT availability measured with PET.

## Material and Methods

### Participants

Ten Caucasians with severe obesity, otherwise healthy, non-depressive (8 females, 2 males; BMI: 46.9 ± 4.9 kg/m^2^, BW: 134.6 ± 18.7 kg; age: 43.9 ± 13.1 years; 1 dropout for post-surgery data) undergoing RYGB were included in this study. They were screened for exclusion criteria, such as neurological or psychiatric disorders as assessed by structured clinical interview; head trauma or vascular encephalopathy; malignant hypertension; insulin-dependent diabetes; anorectic medication or other interventions for weight loss; other chronic diseases that necessitate centrally acting medication; over-the-counter medication or nutrition supplements during the last 2 months; past or present history of alcohol or drug abuse; pregnancy and breast-feeding; and general contraindications for magnetic resonance imaging (MRI) or PET. After obtaining written informed consent, the use of illicit drugs by urine testing and the presence of an eating disorder were excluded by using the German version of the Eating Disorder Examination (EDE) [19], respectively. In addition, all participants received a general physical examination, including height and BW measurements for BMI calculation. The amount of alcohol and/or nicotine consumption was documented. To assess eating behavior, study participants filled in the German language version of the Three-Factor Eating Questionnaire (TFEQ: *Fragebogen zum Essverhalten*, FEV), where upon we focused on the subscale *hunger* [20] and the Beck Depression Inventory (BDI) [21] to screen for depression.

### Imaging and Data Acquisition

The study participants underwent MRI and PET with [^11^C]MRB for baseline data (pre-surgery; *n* = 10) 2 weeks before RYGB surgery and another PET scan 6 months after RYGB surgery (32 weeks ± 2.5; post-surgery; *n* = 9) data. MRI data were acquired by using a 3 Tesla Siemens scanner (Erlangen, Germany) and a T1-weighted 3D magnetization prepared rapid gradient echo (MP-RAGE) sequence (time of repetition 2300 ms, time of echo 2.98 ms, 176 slices, field of view 256 × 240 mm, voxel size 1 × 1 × 1 mm) for PET-MRI data co-registration. Radiotracer was synthesized based on standardized procedures [22]. Dynamic PET imaging (23 frames: 4 × 0.25 × 1.5 × 2.5 × 5.5 × 10 min) was performed with data acquisition starting immediately with intravenous bolus (90 s) injection of 482 ± 31 MBq [^11^C]MRB for pre- and post-surgical scan, by using an ECAT EXACT HR + scanner (Siemens, Erlangen, Germany; intrinsic resolution at the center: 4.3 mm; axial resolution: 5–6 mm; field of view: 15.5 cm, 3–4 mm full width at half maximum) in 3D acquisition mode over 90 min. Ten minutes before emission scanning, a transmission scan with three ^68^Ge sources was performed for attenuation correction. Data were iteratively reconstructed (10 iterations, 16 subsets) in transverse image series (63 slices, 128 × 128 matrix, voxel size 2.6 × 2.6 × 2.4 mm) with a Hann filter (cutoff 4.9 mm).

MRI and PET data were coregistered after spatially reorientation of each MRI data set by rigid transformation onto a standard brain data set (anterior commissure–posterior commissure line), similar to the Talairach space (PMOD 3.5, PMOD Technologies, Zurich, Switzerland). PET scans were motion corrected using SPM2 software (Statistical Parametric Mapping; Wellcome Trust, Centre for Neuroimaging, London, UK). Further, we used PMOD to integrate hand-drawn volumes of interest (VOIs) from a standard VOI map into the MRI data sets. These VOIs include the medial prefrontal cortex (MPFC), dorsolateral prefrontal cortex (DLPFC), anterior cingulate cortex (ACC), orbitofrontal cortex (OFC), insula, head of caudate, putamen, thalamus, hypothalamus, hippocampus, LC, raphe dorsalis, nucleus ruber (NR), and the cerebellar cortex (CC). The individual VOI set was matched with the corresponding PET scan via PMOD, to obtain tissue time activity curves (TACs) from dynamic PET scan. Kinetic modeling was performed using the multilinear reference tissue model MRTM2 (two parameters) with the occipital cortex as reference region [23] to estimate the non-displaceable binding potential (BP_ND_) and the distribution volume ratios (DVR = BP_ND_ + 1). The DVR in occipital cortex was constant over the measurements (*p* = 0.55).

### Statistics

Statistic calculations were done using SPSS (version 24, SPSS Inc. IBM, Armonk, NY, USA). First, all data were tested for normal distribution using Kolmogorov–Smirnov test. In case of normal distribution, we applied Pearson’s correlation and Spearman’s rho in case of not normally distributed data, concerning DVR for pre-surgical ACC, post-surgical insula, and hippocampus, each on the right-hand side. We applied post hoc test for multiple testing. Correlations keeping significance afterwards are entitled as Bonferroni corrected. Differences in normally distributed data were tested using Student’s *t* test and in not normally distributed data using the Mann–Whitney test. All the delta (Δ) data used here were created by subtracting post-surgical data from the pre-surgical data.

## Results

### Changes of BMI/BW from Pre- to Post-surgery

**Fig. 1:**
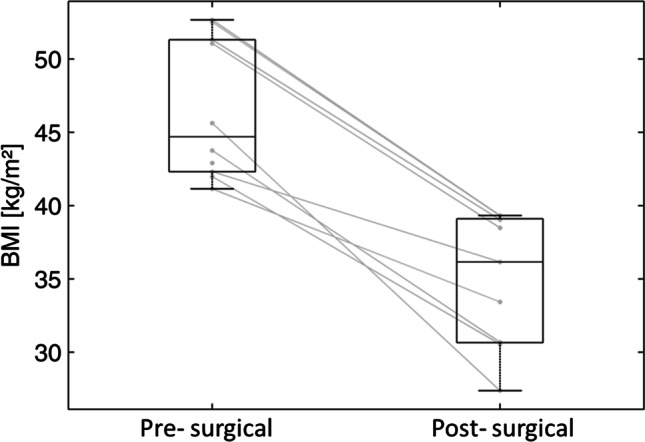
BMI before and 6 months after RYGB surgery (*n* = 9)

After 6 months, all participants responded on RYGB surgery with a mean reduction in BMI of 12.0 ± 3.3 kg/m^2^ (pre-surgical BMI: 46.9 ± 4.8 kg/m^2^; post-surgical BMI: 34.9 ± 4.6 kg/m^2^; *p* = 0.001; Fig. 1). This Δ BMI corresponds to an average BW loss of 35.4 ± 11.7 kg (*p* = 0.001). There is no correlation between the pre-surgical BMI and Δ BMI (*r* = 0.45, *p* = 0.23).

### Changes of DVR from Pre- to Post-surgery

**Fig. 2:**
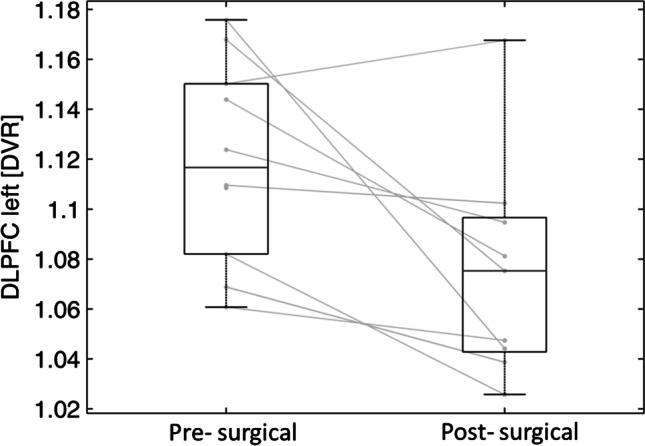
Distribution volume ratios (DVR) of dorsolateral prefrontal cortex (DLPFC) before (pre-surgical) and 6 months after (post-surgical) RYGB (*n* = 9)

**Fig. 3:**
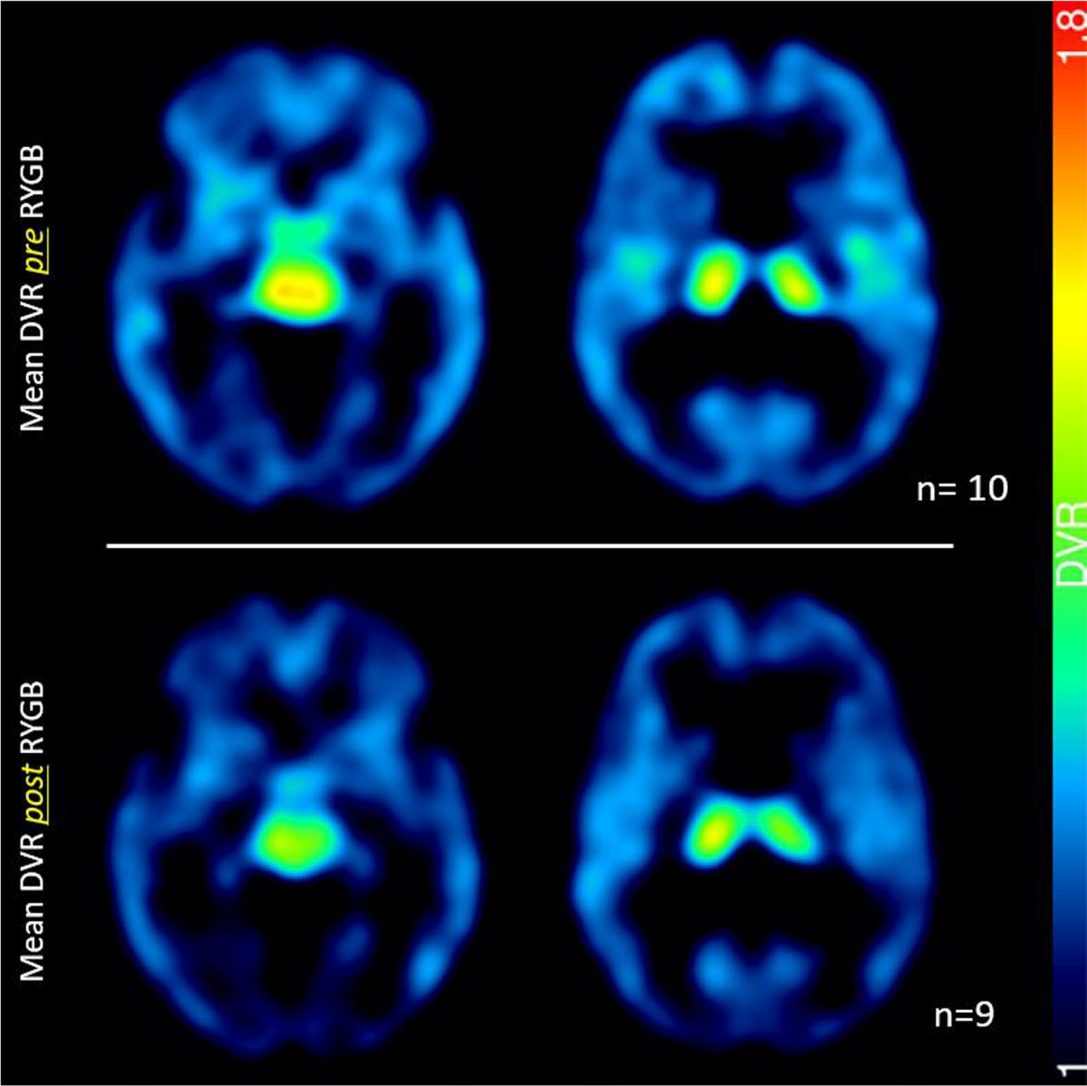
Comparison of mean distribution volume ratios (DVR) before (*n* = 10) and 6 months after RYGB (*n* = 9)

DVR in the DLPFC significantly decreased from 1.12 ± 0.04 to 1.07 ± 0.04 (*p* = 0.019) (Fig. 2). Furthermore, we found a borderline significance for left hypothalamus with decrease from 1.45 ± 0.18 to 1.27 ± 0.19 (*p* = 0.06). No other significant changes were found despite an overall trend towards lowered DVR in post-surgical measurements, as mean DVR comparison shows (Table 1, Fig. 3).

**Table1:** Comparison of distribution volume ratios (DVR; occipital cortex as reference region) of pre- and post-surgical data (*n*=9)

Brain region		Mean DVR ± SD pre	Mean DVR ± SD post	*p* (paired *t* test)
Medial prefrontal cortex	r	1.07 ± 0.05	1.07 ± 0.04	0.67
	l	1.06 ± 0.04	1.05 ± 0.07	0.44
Dorsolateral prefrontal cortex	r	1.11 ± 0.04	1.07 ± 0.08	0.07
	**l**	**1.12 ± 0.04**	**1.07 ± 0.04**	**0.02**
Orbitofrontal cortex	r	1.04 ± 0.08	1.04 ± 0.12	0.25
	l	1.09 ± 0.06	1.05 ± 0.11	0.30
Anterior cingulate cortex	r	1.16 ± 0.07	1.10 ± 0.14	0.14
	l	1.18 ± 0.07	1.14 ± 0.11	0.27
Insula	r	1.23 ± 0.06	1.20 ± 0.08	0.17
	l	1.20 ± 0.08	1.17 ± 0.13	0.37
Hippocampus	r	1.10 ± 0.08	1.04 ± 0.17	0.31
	l	1.12 ± 0.06	1.08 ± 0.18	0.53
Head of the caudate	r	1.14 ± 0.10	1.11 ± 0.07	0.29
	l	1.15 ± 0.07	1.07 ± 0.13	0.16
Putamen	r	1.19 ± 0.15	1.13 ± 0.18	0.33
	l	1.21 ± 0.11	1.20 ± 0.09	0.44
Thalamus	r	1.56 ± 0.18	1.53 ± 0.20	0.61
	l	1.55 ± 0.16	1.45 ± 0.22	0.10
Hypothalamus	r	1.44 ± 0.12	1.32 ± 0.29	0.36
	l	1.45 ± 0.18	1.27 ± 0.19	0.06
Cerebellar cortex	r	1.08 ± 0.05	1.05 ± 0.03	0.12
	l	1.08 ± 0.05	1.07 ± 0.05	0.22
Locus coeruleus		0.42 ± 0.17	0.29 ± 0.30	0.17
Raphe dorsalis		0.51 ± 0.17	0.42 ± 0.21	0.38

### Association Between DVR and BMI

Pre-surgical BMI and the pre-surgical DVR were significantly associated in the OFC (right, *r* =  − 0.76, *p* = 0.01; left, *r* =  − 0.77, *p* = 0.01), the CC (*r* =  − 0.81, *p* = 0.005), the left ACC (*r* =  − 0.66, *p* = 0.037), and the left DLPFC (*r* = 0.70, *p* = 0.024) (Table 2). There were no significant correlations between the changes in BMI (Δ BMI) and the changes in DVR (Δ DVR).

**Table 2:** Correlations (Pearson’s correlation coefficient *r*) between pre-surgical DVR and pre-surgical BMI (*n* = 10) as well as with change in BMI (Δ BMI) over 6 months after RYGB (*n* = 9)

Brain regions (DVR)		*BMI* (*r*)	*p*	*Δ BMI* (r)	*p*
Medial prefrontal cortex	r	− 0.097	0.79	0.140	0.72
	l	− 0.036	0.92	0.416	0.27
Dorsolateral prefrontal cortex	r	− 0.449	0.19	− 0.186	0.63
	**l**	**0.700**	**0.02**	0.586	0.10
Orbitofrontal cortex	**r**	− **0.757**	**0.01**	− 0.224	0.56
	**l**	− **0.765**	**0.01**	− 0.460	0.21
Anterior cingulate cortex	r	− 0.410	0.24	− 0.331	0.39
	**l**	− **0.662**	**0.04**	− 0.540	0.13
Insula	r	− 0.476	0.16	0.203	0.60
	l	− 0.511	0.13	− 0.077	0.84
Hippocampus	r	− 0.431	0.21	− 0.637	0.07
	l	− 0.258	0.47	0.122	0.76
Head of the caudate	r	− 0.188	0.60	− 0.100	0.80
	**l**	0.332	0.35	**0.838**	**0.005**
Putamen	**r**	0.062	0.86	− **0.736**	**0.02**
	**l**	− 0.135	0.71	− **0.818**	**0.007**
Thalamus	r	0.157	0.67	− 0.176	0.65
	l	0.077	0.83	− 0.099	0.80
Hypothalamus	**r**	0.361	0.31	**0.778**	**0.014**
	l	− 0.517	0.13	− 0.374	0.32
Cerebellar cortex	r	− 0.433	0.21	− 0.217	0.58
	**l**	− **0.809**	**0.01**	− 0.490	0.18
Locus coeruleus		0.339	0.34	− 0.191	0.62
Raphe dorsalis		0.009	0.98	0.463	0.21

### Pre-surgical DVR and Changes in BMI from Pre-surgery to 6-Month Follow-Up

**Fig. 4:**
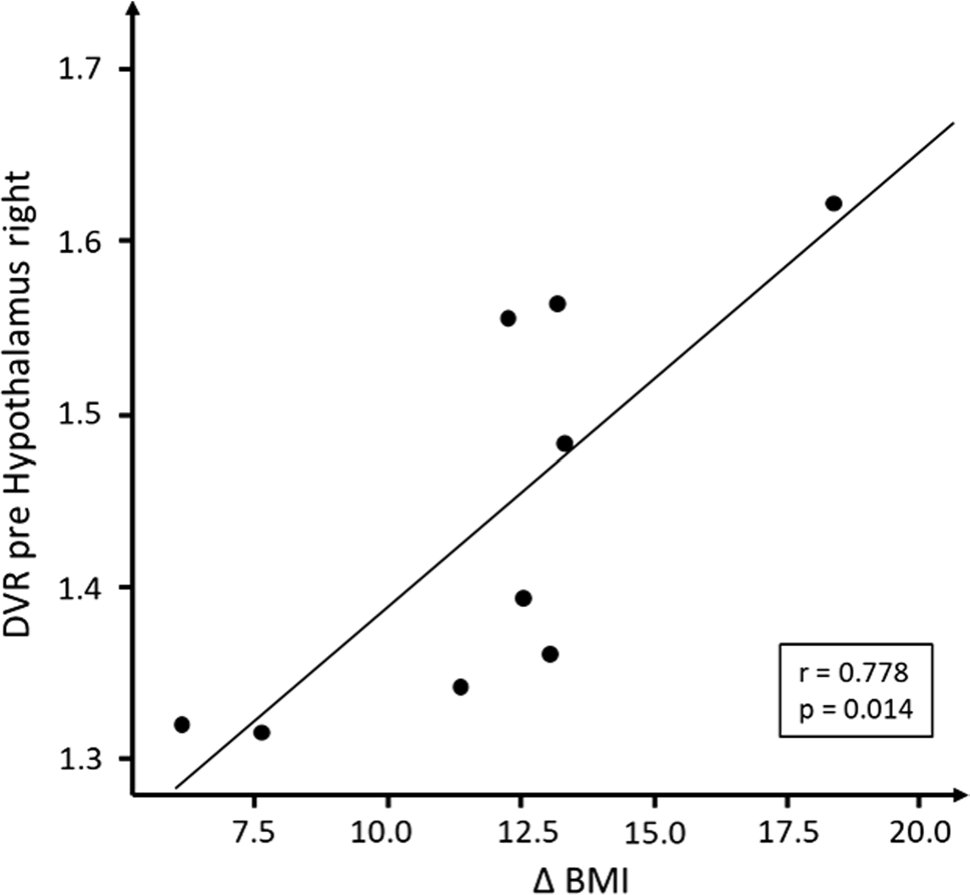
Correlation between pre-surgical distribution volume ratios (DVR) and changes in body mass index (ΔBMI) (*n* = 9). This positive correlation indicates a relationship between a pre-surgical DVR and BMI reduction, i.e., the higher the pre-surgical DVR, the greater the decrease in BMI

We found significant positive correlations between the pre-surgical DVR and Δ BMI in the right hypothalamus (*r* = 0.78; *p* = 0.014) (Fig. 4) and the left head of the caudate (*r* = 0.84, *p* = 0.005). Negative correlations were found in the putamen (right, *r* =  − 0.74, *p* = 0.02; left, *r* =  − 0.82, *p* = 0.007) (Table 2).

### Association of DVR and TFEQ

**Fig. 5:**
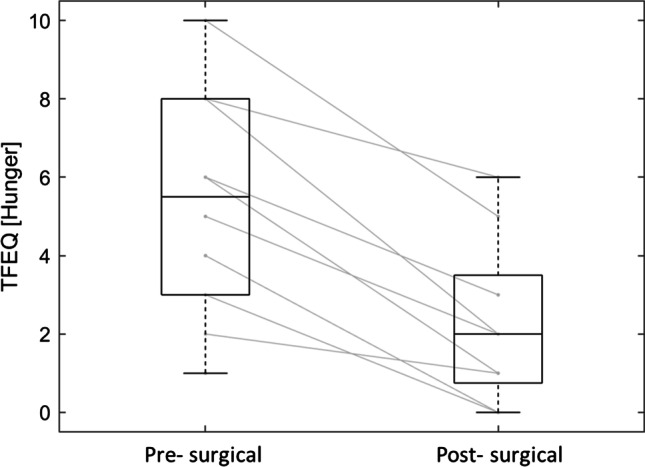
Three-Factor Eating Questionnaire (TFEQ) hunger subscale scores before (pre-surgical) and 6 months after (post-surgical) RYGB surgery (*n* = 10)

**Fig. 6:**
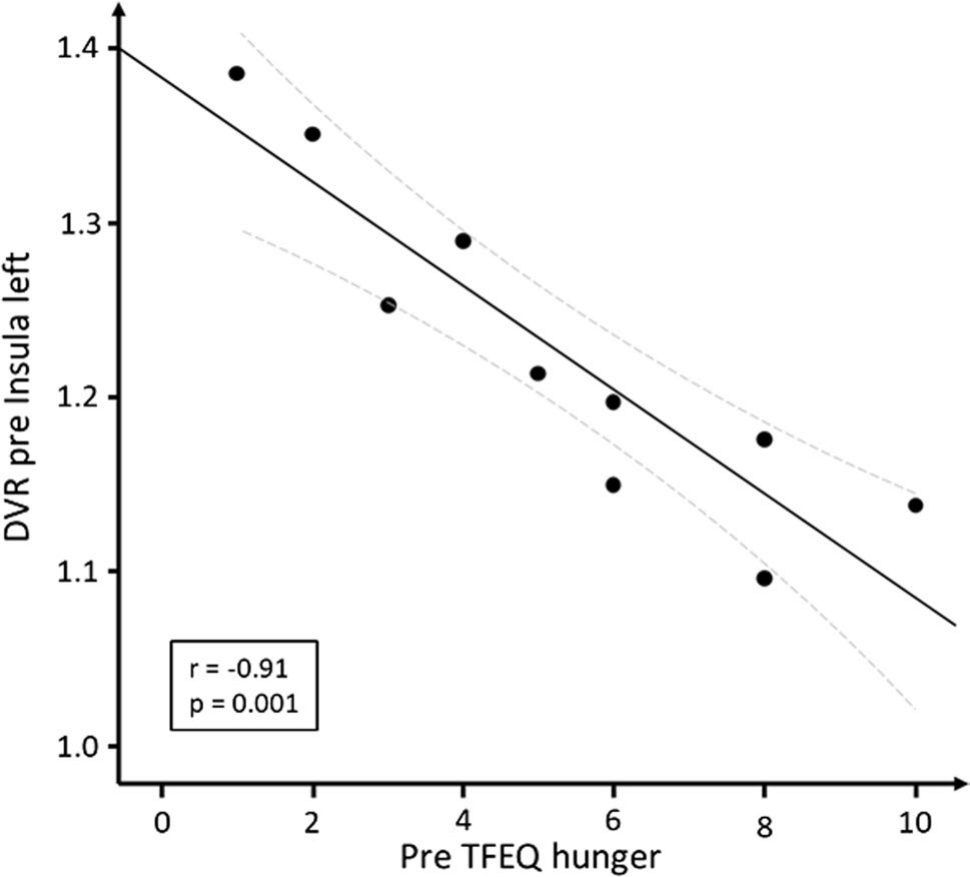
Correlation between pre-surgical distribution volume ratios (DVR) of left insula and pre-surgical scores TFEQ hunger subscale (*n* = 10, curved lines show 95% confidence interval)

TFEQ hunger subscale significantly declined between pre- and post-surgical measurements (Fig. 5). Further, we found a strong negative correlation between the pre-surgical DVR of the left and of the right insula and the pre-surgically derived scores of the TFEQ hunger subscale (*r* =  − 0.91, *p* = 0.0024, and *r* =  − 0.87, *p* = 0.024, respectively, Bonferroni corrected; Fig. 6). No other significant correlations between any brain region and the TFEQ were found.

## Discussion

Despite being the most effective treatment for severe obesity, the understanding of how RYGB affects central neurotransmission, specifically the function of brain monoamines, is still at its beginning. Own previous study using PET and the NAT-selective radiotracer [^11^C]MRB indicated that NAT availability is a marker of the integrity of the LC-NA system, and that its tone is altered in relation to changes in BMI and BW [16, 18, 24]. Based on these data, we hypothesized that NAT availability changes 6 months after RYGB surgery in relation to changes in BMI. Specifically, we expected a reduction in NAT availability post-surgery in brain areas relevant for inhibitory and homeostatic control, i.e., the DLPFC and the hypothalamus, respectively, compared with pre-surgery.

As expected, we found that together with a substantial loss of BW at 6-month follow-up, there was a significant decrease in NAT availability in the DLPFC. This finding is in agreement with a recent fMRI investigation showing that neuronal activity in the DLPFC has predictive power for future weight loss [25]. By extension, neuronal activity in DLPFC might serve as a measurable surrogate not only for post-diet weight gain or loss, but also for predicting the success of RYGB surgery. Indeed, patients with greater weight loss after RYGB have greater activation of the DLPFC in a resist task to images of appetizing foods [26].

We also found that NAT availability in the hypothalamus correlated highly with changes in BMI. That is, in our cohort, high pre-surgical NAT availability in the hypothalamus was associated with greater weight loss after 6 months and vice versa. However, NAT availability in the hypothalamus was not affected by RYGB. This is in line with a previous PET study with [^11^C]MRB showing that obesity is not associated with changes in NAT availability in the hypothalamus [18]. Rodent studies have shown that microinjection of NA into the paraventricular nucleus of the hypothalamus increases food intake and body weight [27] and that NA elicits a profile of neuronal activity in the arcuate nucleus of the hypothalamus that promotes feeding [28]. Future studies using rodent models of RYGB can reveal how high hypothalamic NAT availability before surgery relates to weight loss after surgery and to local noradrenergic tone.

Besides this finding, the correlations between the putamen and changes in BMI confirm perceptions of former fMRI-based investigations on food cue reactivity. Multiple studies show strong dependency between response in fMRI scans due to high-calorie food images performed prior to [29], or early during intervention [30] and definitive success in body weight reduction after treatment. In an analogical study, comparable effects on the OFC have been reported.

In our brief psychological assessment, we additionally found a highly significant correlation between pre-surgical NAT availability in the insula and the subjective feeling of hunger, measured by the corresponding subscale of the TFEQ. This finding corroborates our previous PET investigation in a separate cohort of patients undergoing treatment-as-usual to reduce BW [17], also showing a strong correlation between the left insula cortex and hunger measured by the TFEQ. It is an interesting result since the insula is involved in numerous functions and neural circuits that cover somatosensory functions, as well as pain and temperature, but also taste and olfactory perception. Further, the insular cortex receives homeostatic afferents, concerning information of physiological conditions such as thirst and hunger, and integrates them with emotional evaluation [31].

### Limitations

The study was conceptualized as a proof-of-investigation to initiate forthcoming confirmatory studies. Therefore, the sample size of our cohort is too small to draw definite conclusion. Furthermore, the post-surgical observation period of 6 months might also be too short to depict the final adaption on neurotransmitter level. In order to give definitive statement on changes on central NA levels, we therefore need longer follow-up periods and balanced distribution in sex among participants as well. The radiotracer [^11^C]MRB applied here only allows indirect measurement of synaptic and volume NA transmission. Yet, the relationship between noradrenergic tone and NAT presence is unclear. So, lowered availability in NAT could be related to lower NA reuptake and therefore to a higher extracellular concentration of NA, or NAT may be lowered resulting from a lowered NA release into the extracellular space and generally low NA activity. Furthermore, the radiotracer itself has limitations concerning low BP [18]. We applied the currently most effective treatment approach for obesity, from which we expected significantly pronounced effects of RYGB surgery on BMI. Associated with this, we would expect measurable effects on the central noradrenergic system after RYGB. Altogether, for the confirmation of our findings, further investigations with larger cohorts and longer observation periods are necessary and will lead to deeper understanding of alterations on NA system following RYGB but also other treatments targeting BW loss.

### Conclusions

Our pilot data suggest that changes of BMI 6 months after RYGB surgery are associated with changes of NAT availability in specific brain regions responsible for appetite control and energy homeostasis. These first results need further verification in larger series with longer follow-up periods in order to assess whether NAT availability as measured with PET prior RYGB surgery has the potential to serve as a biomarker to prognosticate the outcome of RYGB on BMI.
